# Optimization of High-Pressure Ultrasonic-Assisted Simultaneous Extraction of Six Major Constituents from *Ligusticum chuanxiong* Rhizome using Response Surface Methodology

**DOI:** 10.3390/molecules19021887

**Published:** 2014-02-10

**Authors:** Jin-Liang Liu, Shun-Lin Zheng, Qiao-Jia Fan, Ji-Chao Yuan, Shi-Min Yang, Fan-Lei Kong

**Affiliations:** 1 College of Agronomy, Sichuan Agricultural University, Chengdu 611130, China; E-Mails: liujinliang0827@163.com (J.-L.L.); zhengshunlin123@163.com (S.-L.Z.); yangshimin1@163.com (S.-M.Y.); kongfanlei@sicau.edu.cn (F.-L.K.); 2 Key Laboratory of Crop Ecophysiology and Farming System in Southwest China, Ministry of China, Chengdu 611130, China; 3 College of Veterinary Medicine, Sichuan Agricultural University, Ya’an 625014, China; E-Mail: fanqj@live.cn

**Keywords:** *Ligusticum chuanxiong*, major constituents, response surface methodology, optimization, high-pressure ultrasonic-assisted extraction

## Abstract

High-pressure ultrasound-assisted extraction technology was applied to extract ferulic acid, senkyunolide I, senkyunolide H, senkyunolide A, ligustilide and levistolide A from *Ligusticum chuanxiong* rhizomes. Seven independent variables, including solvent type, pressure, particle size, liquid-to-solid ratio, extraction temperature, ultrasound power, and extraction time were examined. Response Surface Methodology (RSM) using a Central Composite Design (CCD) was employed to optimize the experimental conditions (extraction temperature, ultrasonic power, and extraction time) on the basis of the results of single factor tests for the extraction of these six major components in *L. chuanxiong* rhizomes. The experimental data were fitted to a second-order polynomial equation using multiple regression analysis and were also examined using appropriate statistical methods. The best extraction conditions were as follows: extraction solvent: 40% ethanol; pressure: 10 MPa; particle size: 80 mesh; liquid-to-solid ratio: 100:1; extraction temperature: 70 °C; ultrasonic power, 180 W; and extraction time, 74 min.

## 1. Introduction

Chuanxiong Rhizoma, the dried rhizome of *Ligusticum chuanxiong* Hort. (Umbelliferae), is a herb that has been widely used in Traditional Chinese Medicine for a long time [1]. It is frequently prescribed to treat angina pectoris, cardiac arrhythmias, hypertension, and stroke because it helps in blood circulation and disperses blood stasis [2,3]. The essential biologically active components of *L. chuanxiong* are organic acids, alkaloids, and phthalides [4,5]. 

Extraction of herbs for Traditional Chinese Medicines has often been a good choice for process engineers in production development and for product quality evaluation. To date, several conventional extraction techniques, such as decoction [1,6], percolation [6,7], sonication [7,8,9], reflux [5,10,11,12,13,14,15,16,17,18,19,20], and microwave-assisted extraction [21,22], have been reported for the extraction of ferulic acids and/or phthalides from Chuanxiong Rhizoma. However, these extraction methods, summarized in Table 1, are expensive [21], have low efficiency [5,6,8,9,10,11,12,13,16,17,18,19,20,21,22], require long extraction times [5,6,7,10,11,12,13,14,15,17,18,19,20,21], and/or high temperatures [5,6,10,11,14,15,17,19]. Moreover, the conditions used in these methods are not consistent, and in many cases one cannot determine how these conditions were established and optimized, and there are no reports on systematic optimization of the extraction conditions for multiple ingredients in *L. chuanxiong*. 

With the development of the “green chemistry” concept during the past decade, environmentally friendly extraction techniques are becoming more popular. Ultrasound-assisted extraction is a new technique that has attracted much attention in natural chemical product extraction in recent years. Ultrasound is usually generated by a transducer that converts mechanical or electrical energy into high frequency vibrations and produces cavitation in the solvent by the passage of ultrasonic waves. This technique offers high reproducibility with short extraction times and lower consumption of solvents, temperatures and energy inputs [23,24,25,26,27,28,29]. Various factors such as solvent type, material particle size, liquid-to-solid ratio, extraction temperature, extraction time, and ultrasonic power [30,31,32,33] could all affect the ultrasound-assisted extraction of antioxidants, polysaccharides, and phenolic compounds. Pressure has also been found to affect the extraction yield of active ingredients [34]. Sun and Wang analyzed the influence of extraction solvent types, ultrasonic power, extraction temperature, and extraction time on the extraction yield of ferulic acid in *L. chuanxiong* [9]. However, pressure has not been reported as a factor in the ultrasonic-assisted simultaneous extraction of the six major components *L. chuanxiong* (ferulic acid, senkyunolide I, senkyunolide H, senkyunolide A, ligustilide and levistolide A).

Optimization of a process is generally achieved either through empirical or statistical methods. The empirical method does not reveal the complete effects of each parameter on the response. Moreover, this method increases the number of experiments needed to conduct the study, time and expense and the amounts of reagents and materials consumed. Response Surface Methodology (RSM) enables the evaluation of the effects of the variables and their interactions with the response variables [35,36,37,38]. RSM is a collection of statistical and mathematical techniques that has been extensively used for developing, improving, and optimizing extraction conditions [39,40,41,42,43]. The this article we report the first application of high-pressure ultrasound-assisted extraction combined with RSM to the optimization of the extraction of the main active ingredients of Chuanxiong Rhizoma.

**Table 1:** Extraction conditions of different methods used for *L. chuanxiong* rhizoma.

No.	Constituents	Method	Solvent	Time (min)	Temperature (°C)	Extraction yield (%)	Ref.
1	Ferulic acid	Ultrasound	4 mL ethanol	30	25	0.06	[9]
2	Ferulic acid	Reflux	12 mL 75% ethanol	270	100	0.13	[10]
3	Ferulic acid	Reflux	8 mL 70% ethanol	180	100	0.11	[11]
4	Ferulic acid	Reflux	7.5 mL 80% ethanol	180	Unclear	0.11	[12]
5	Ferulic acid	Percolation and decoction	12 mL water	180	100	0.05–0.06	[6]
6	Ferulic acid	Reflux	6 mL 90% ethanol	358	Unclear	0.14	[13]
7	Ferulic acid	Microwave	10 mL 40% ethanol	4	Unclear	0.08	[22]
8	Ferulic acid	Reflux	54 mL 40% ethanol	180	100	0.10–0.19	[14]
9	Ferulic acid	Reflux	5 mL 90% ethanol	90	100	0.11–0.18	[15]
10	Ferulic acid	Reflux	5 mL water	60	50	0.05	[16]
11	Ferulic acid	Reflux	8 mL 70% ethanol	180	100	0.11	[17]
12	Ferulic acid Ligustilide	Reflux	15 mL 80% ethanol	180	Unclear	0.35–0.12; 0.07–0.20	[18]
13	Ligustilide	Reflux	25 mL 71% ethanol	278	89	0.68	[19]
14	Senkyunolide A Ligustilide	Distillation	water	540	100	0.04; 0.21	[5]
15	Senkyunolide I Senkyunolide H Ligustilide	Microwave	15 mL ionic liquid	5	180	about 0.08; about 0.02; about 0.40	[21]
16	Senkyunolide I Senkyunolide H Ligustilide	Reflux	5 mL 75% ethanol	270	60	0.10–0.20; 0.05–0.09; 0.44–0.53	[20]
17	Ferulic acid Senkyunolide I Senkyunolide H Ligustilide Levistolide A	Percolation and ultrasound	25 mL ethanol	>300	Unclear	0.51; 1.32; 0.47; 3.74; 0.05	[7]

*Note*: 0.5 g sample to calculate the volume of extraction solvent.

In this study, seven factors, namely solvent type, pressure, particle size, liquid-to-solid ratio, extraction temperature, ultrasound power, and extraction time, were investigated first to optimize the extraction solvent, pressure, particle size, and liquid-to-solid ratio. Additionally, the levels of the response surface experimental design factors (temperature, power, and time) were determined according to the extraction temperature, ultrasonic power, and extraction time optimized in the single factor tests.

## 2. Results and Discussion

### 2.1. Analysis of Single Factor Test Results

#### 2.1.1. Effect of Solvent Type on Extraction

**Figure 1:**
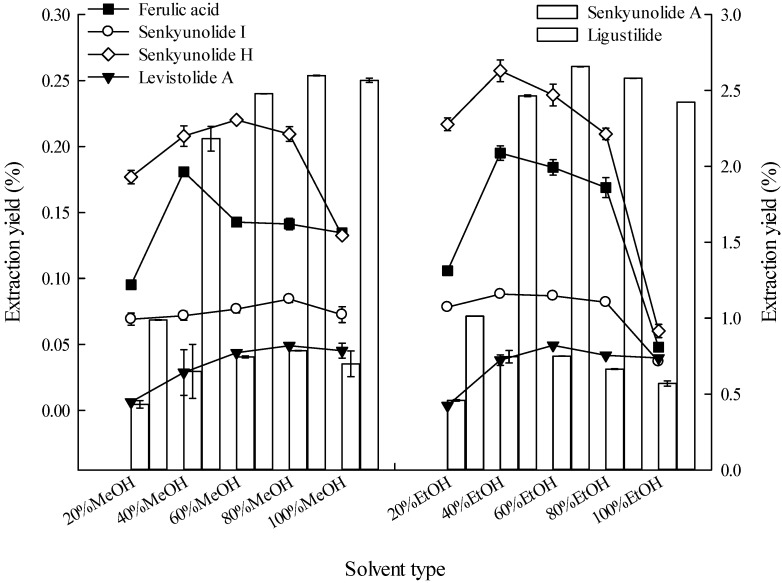
Effects of solvent type on the extraction yields of the six constituents (the lines and bar graphs as referenced to the left and right axes, respectively).

Solvent selection is important in the extraction of compounds from botanical materials [30]. Figure 1 shows that under similar extraction conditions 40% EtOH is superior to other solvents in extracting ferulic acid and senkyunolide H from *L. chuanxiong* rhizome. Both 40% EtOH and 60% EtOH are superior to the other solvents in extracting senkyunolide I, but without significant differences in their extraction yields (analysis of variance, *p* ≤ 0.05, the same below). Additionally, 80% MeOH and 60% EtOH are superior to other solvents in extracting levistolide A, but not significantly different from 40% EtOH. Moreover, 80% MeOH and 40% EtOH are superior to other solvents in extracting senkyunolide A, but without significant differences. The 60% EtOH solvent is superior to other solvents in extracting ligustilide and does not significantly differ from 40% EtOH.

Ethanol is a Good Manufacturing Practice-compliant solvent. Based on the results, 40% ethanol was selected as the extraction solvent to extract the six major constituents using high-pressure ultrasonic-assisted extraction in subsequent experiments. Previous studies have reported the use of ethanol (mainly for extracting ferulic acid) [1,7,9,10,11,12,13,14,15,17,18,19,20,22], water [5,6,16], methanol [8,44], and protic ionic liquids [21] as extraction solvents for extracting ingredients from *L. chuanxiong* rhizomes. However, methanol is toxic, water is unable to dissolve the hydrophobic components, protic ionic liquids only extracted three lactone constituents, so although the ethanol concentrations using the above method were somewhat variable, ethanol was chosen as the best extraction solvent among the extraction solvents listed in Section 3.3.1.

#### 2.1.2. Effect of Pressure on Extraction

**Figure 2:**
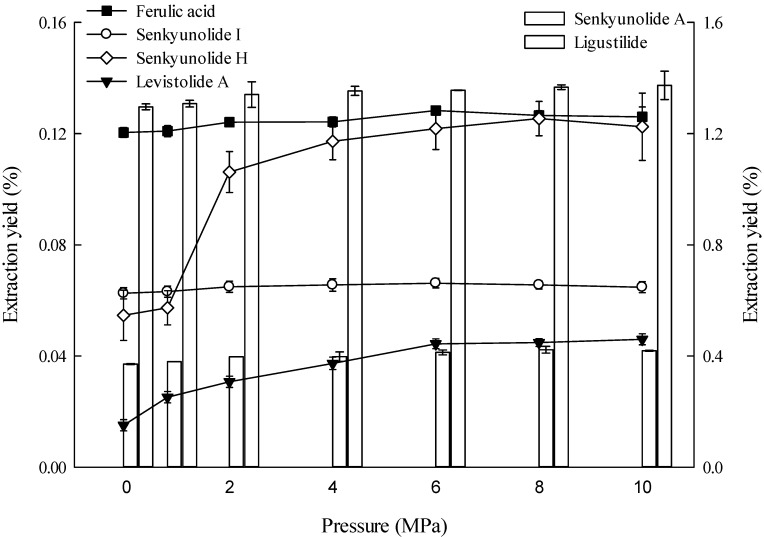
Effects of pressure on extraction yields of the six constituents (the lines and bar graphs are referenced to the left and right axes, respectively).

The high pressure that promotes ultrasonic cavitation also creates shear forces that break cell walls mechanically. The cavitation bubbles from the increased pressure causes the solvent to penetrate deeper into the raw plant material/intracellular space [34,45]. Figure 2 presents how different pressure levels affect the extraction yield of the six major constituents in *L. chuanxiong*. Pressures of 8 and 10 MPa are superior to other pressure levels in extracting senkyunolide H and levistolide A, but the extraction yields between these two pressures do not differ significantly. The extraction yields of ferulic acid, senkyunolide I, senkyunolide A, and ligustilide continued to increase with increasing pressure and do not significantly differ between 4 MPa to 10 MPa. 

#### 2.1.3. Effect of Particle Size on Extraction

**Figure 3:**
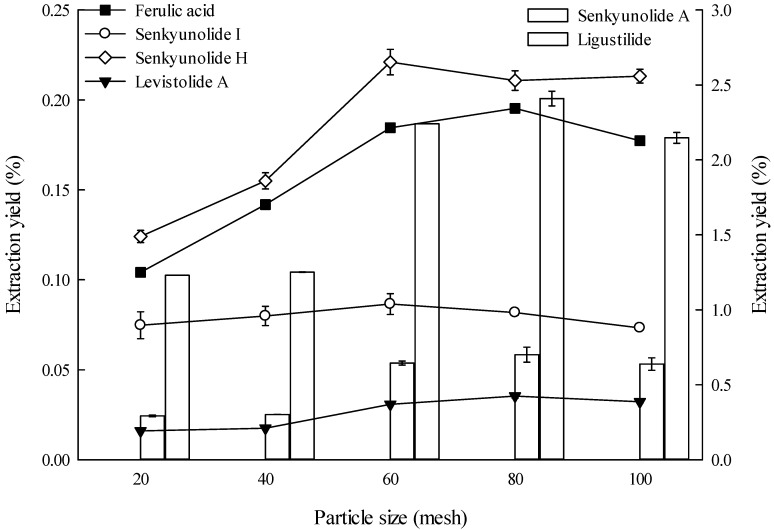
Effects of particle size on the extraction yields of the six constituents (the lines and bar graphs are referenced to the left and right axes, respectively).

The appropriate particle size is fundamental to obtain optimal extraction, and varied particle sizes could significantly affect the extraction yield. In this study, 80 mesh was better than other particle sizes in extracting ferulic acid, levistolide A, senkyunolide A, and ligustilide (Figure 3), whereas 60 mesh was better than other particle sizes in extracting senkyunolide I and senkyunolide H. These two mesh sizes do not differ significantly.

**Figure 4:**
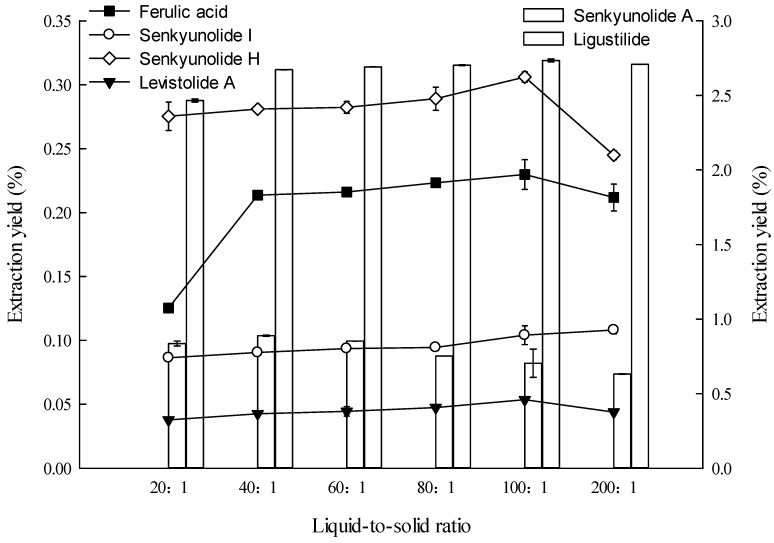
Effects of liquid-to-solid ratio on the extraction yields of the six constituents (the lines and bar graphs are referenced to the left and right axes, respectively).

#### 2.1.4. Effect of Liquid-to-Solid Ratio on Extraction

For efficient extraction, the solvent volume must be sufficient to ensure complete immersion of materials, and an extraction solvent deficiency can lead to lower extraction yields (incomplete extraction) of ingredients [9,10,11,12,15,17,18,20,22], but redundant solvent may also lead to lower extraction yields and solvent waste [31]. Therefore, the liquid-to-solid ratio must be appropriate. The effect of liquid-to-solid ratio on the extraction yield of the six constituents was investigated, and the results are shown in Figure 4. The 100:1 (v/m) proportion is better than other liquid-to-solid ratios in extracting ferulic acid, senkyunolide H, ligustilide, and levistolide A. The 100:1 and 200:1 proportions are better than other liquid-to-solid ratios in extracting senkyunolide I, but without a statistically significant difference. The 40:1 ratio is better than other ratios in extracting senkyunolide A. To minimize solvent requirements and without compromising responses, the liquid-to-solid ratio of 100:1 (v/m) was selected. 

#### 2.1.5. Effect of Temperature on Extraction

**Figure 5:**
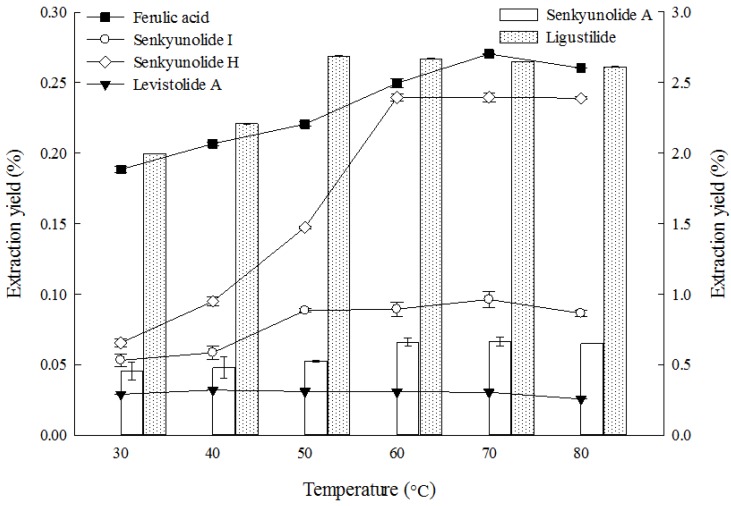
Effect of temperature on the extraction yields of the six constituents (the lines and bar graphs are referenced to the left and right axes, respectively).

The effect of temperature on the extraction yield of the six main constituents from *L. chuanxiong* rhizome was investigated, and the results are shown in Figure 5. 

#### 2.1.6. Effect of Ultrasonic Power on Extraction

**Figure 6:**
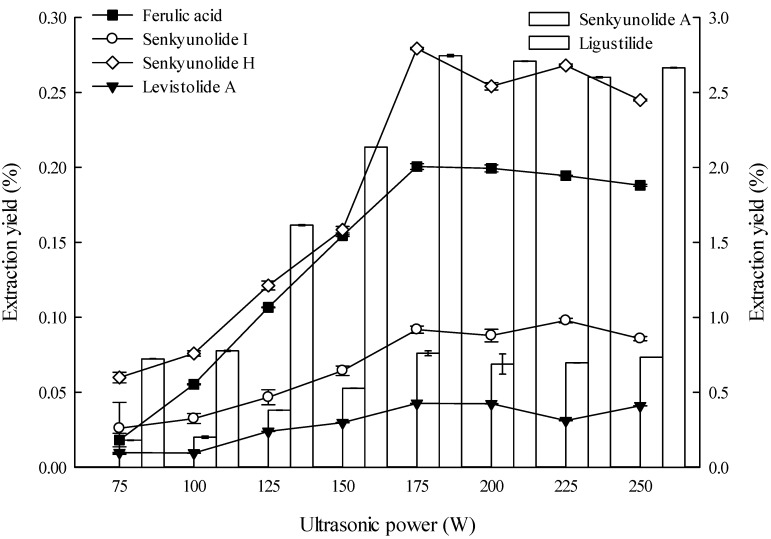
Effect of ultrasonic power on the extraction yields of the six constituents (the lines and bar graphs are referenced to the left and right axes, respectively).

Low ultrasonic power reduces extraction yield. However, excessively high power results in energy wastage. Therefore, the optimal ultrasonic power should be determined. The effect of ultrasonic power on the extraction yield of the six main constituents from the *L. chuanxiong* rhizomes was investigated, and the results are shown in Figure 6. The extraction yields of the six constituents from the rhizomes evidently increased with increasing ultrasonic power, but no increase was observed above 175 W. Few studies on effects of ultrasonic power for extraction constituents of *L. chuanxiong* are available but the extraction yields were very low [9].

#### 2.1.7. Effect of Time on Extraction

**Figure 7:**
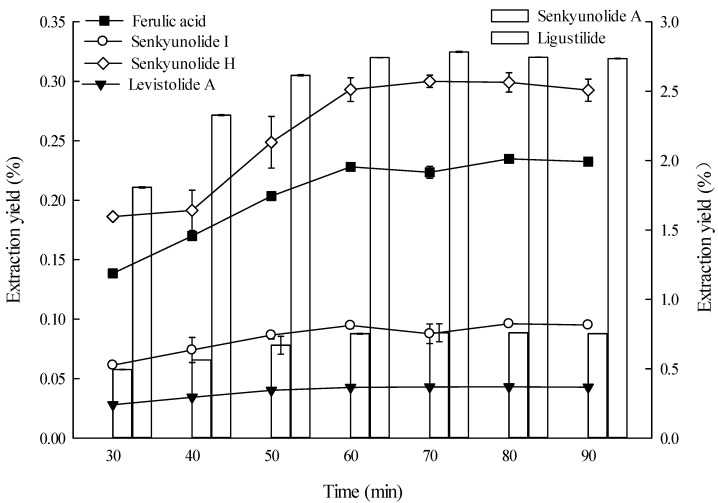
Effect of time on the extraction yields of the six constituents (the lines and bar graphs are referenced to the left and right axes, respectively).

Extraction time, an important parameter in solvent extraction, has two phases, *i.e.*, the dissolution of soluble components on the surfaces of the sample particle and the mass transfer of the solute from the plant matrix into the solvent by diffusion and osmotic processes [24]. The longer the time, solvent and sample contact more fully, and this might accelerate the absorption of solvent, soften the plant tissues and weaken the cell wall integrity, as well as enhance ingredient solubility, thus larger amounts of substances would distribute to the solvent. However, too long a time may lead to lower process efficiency and wasted time. Therefore, the extraction time must be appropriate. The effects of extraction time on the extraction yields of six constituents from *L. chuanxiong* rhizome are shown in Figure 7. The extraction yields of ferulic acid, senkyunolide I and senkyunolide A increased as extraction time was prolonged from 30 min to 60 min and peaked at 60 min. However, the extraction yields of senkyunolide H, levistolide A, and ligustilide increased as the extraction time increased from 30 min to 70 min and peaked at 70 min. 

### 2.2. Model Fitting of Parameters based on the Extraction Yields of the Six Constituents

The responses of the extracts (ferulic acid, senkyunolide I, senkyunolide H, senkyunolide A, ligustilide and levistolide A) in each run are presented in Table 2. The regression coefficients and results from analysis of variance (ANOVA) of the second order polynomial models (Y = A_0_ + A_1_X_1_ + A_2_X_2_ + A_3_X_3_ + A_11_X_1_^2^ + A_22_X_2_^2^ + A_33_X_3_^2^ + A_12_X_1_X_2_ + A_13_X_1_X_3_ + A_23_X_2_X_3_) for ferulic acid, senkyunolide I, senkyunolide H, senkyunolide A, ligustilide and levistolide A are summarized in Table 3. The regression parameters of the surface response analysis of the models, namely, the linear, and quadratic, and their corresponding interaction terms have significant differences (*p* ≤ 0.0001, *p* ≤ 0.01 or *p* ≤ 0.05). The fitness of the model was evaluated through the lack of fit test (*p* < 0.05), which indicates the adequacy of model to predict accurately the variation [46]. The models were used to construct three-dimensional response surface plots to predict the relationship between the independent and dependent variables.

#### 2.2.1. Effect of Process Variables on the Extraction Yield of Ferulic Acid

The experimental data were examined through regression analysis, and the coefficients of the model were evaluated for significance. Temperature (X_1_), ultrasonic power (X_2_), and time (X_3_) significantly affected the extraction yield of ferulic acid (Y_1_, Table 3), with corresponding contribution rates of 1.80, 2.32, and 2.31. These results indicate that ultrasonic power and extraction time have the greatest impact on the extraction yield of ferulic acid.

**Table 2:** Extraction yield of response surface CCD (*n* = 3) and expressed as mean ± SD (Units: %).

Run order	X_1 _(°C)	X_2_ (W)	X_3_ (min)	Ferulic acid	Senkyunolide I	Senkyunolide H	Senkyunolide A	Ligustilide	Levistolide A
1	1(70)	1(200)	1(80)	0.25 ± 0.01	0.09 ± 0.01	0.26 ± 0.01	0.70 ± 0.00	2.65 ± 0.01	0.04 ± 0.01
2	1(70)	1(200)	−1(60)	0.25 ± 0.01	0.09 ± 0.01	0.25 ± 0.03	0.69 ± 0.03	2.63 ± 0.01	0.04 ± 0.01
3	1(70)	−1(150)	1(80)	0.25 ± 0.01	0.09 ± 0.01	0.26 ± 0.01	0.67 ± 0.00	2.63 ± 0.01	0.04 ± 0.01
4	1(70)	−1(150)	−1(60)	0.25 ± 0.01	0.08 ± 0.01	0.25 ± 0.01	0.64 ± 0.00	2.60 ± 0.01	0.04 ± 0.01
5	−1(50)	1(200)	1(80)	0.23 ± 0.01	0.09 ± 0.01	0.26 ± 0.02	0.69 ± 0.05	2.63 ± 0.01	0.04 ± 0.01
6	−1(50)	1(200)	−1(60)	0.23 ± 0.01	0.09 ± 0.01	0.24 ± 0.01	0.68 ± 0.00	2.55 ± 0.01	0.04 ± 0.01
7	−1(50)	−1(150)	1(80)	0.23 ± 0.01	0.06 ± 0.01	0.25 ± 0.01	0.67 ± 0.00	2.57 ± 0.01	0.04 ± 0.01
8	−1(50)	−1(150)	−1(60)	0.12 ± 0.01	0.05 ± 0.01	0.13 ± 0.01	0.42 ± 0.00	1.69 ± 0.01	0.02 ± 0.01
9	−1.682(43)	0(175)	0(70)	0.21 ± 0.01	0.05 ± 0.01	0.17 ± 0.07	0.52 ± 0.07	2.13 ± 0.01	0.03 ± 0.01
10	1.682(77)	0(175)	0(70)	0.28 ± 0.01	0.10 ± 0.01	0.26 ± 0.04	0.67 ± 0.02	2.75 ± 0.01	0.05 ± 0.01
11	0(60)	−1.682(132.5)	0(70)	0.26 ± 0.01	0.08 ± 0.01	0.21 ± 0.01	0.63 ± 0.00	2.29 ± 0.01	0.03 ± 0.01
12	0(60)	1.682(217.5)	0(70)	0.25 ± 0.01	0.09 ± 0.01	0.27 ± 0.01	0.79 ± 0.01	2.69 ± 0.01	0.04 ± 0.01
13	0(60)	0(175)	−1.682(53)	0.21 ± 0.01	0.08 ± 0.01	0.25 ± 0.01	0.58 ± 0.05	2.59 ± 0.01	0.04 ± 0.01
14	0(60)	0(175)	1.682(87)	0.25 ± 0.01	0.09 ± 0.01	0.27 ± 0.01	0.74 ± 0.00	2.82 ± 0.01	0.04 ± 0.01
15	0(60)	0(175)	0(70)	0.24 ± 0.01	0.08 ± 0.01	0.24 ± 0.01	0.64 ± 0.00	2.60 ± 0.01	0.04 ± 0.01
16	0(60)	0(175)	0(70)	0.25 ± 0.01	0.09 ± 0.01	0.25 ± 0.02	0.66 ± 0.06	2.72 ± 0.01	0.04 ± 0.01
17	0(60)	0(175)	0(70)	0.23 ± 0.01	0.09 ± 0.01	0.26 ± 0.01	0.66 ± 0.00	2.57 ± 0.01	0.04 ± 0.01
18	0(60)	0(175)	0(70)	0.24 ± 0.01	0.09 ± 0.01	0.27 ± 0.01	0.68 ± 0.00	2.66 ± 0.01	0.04 ± 0.01
19	0(60)	0(175)	0(70)	0.26 ± 0.01	0.09 ± 0.01	0.29 ± 0.03	0.67 ± 0.03	2.75 ± 0.01	0.04 ± 0.01
20	0(60)	0(175)	0(70)	0.24 ± 0.01	0.09 ± 0.01	0.24 ± 0.08	0.63 ± 0.03	2.71 ± 0.01	0.04 ± 0.01

**Table 3:** Regression coefficients of predicted polynomial models for the investigated responses from *L. chuanxiong* extracts.

Coefficient	Constituents					
	Ferulic acid	Senkyunolide I	Senkyunolide H	Senkyunolide A	Ligustilide	Levistolide A
A_0_	0.245 ***	0.089 ***	0.253 ***	0.658 ***	2.669 ***	0.040 ***
A_1_	0.022 ***	0.011 ***	0.021 **	0.032 **	0.154 **	0.002 **
A_2_	0.013 *	0.007 ***	0.016 **	0.038 **	0.212 **	0.002 **
A_3_	0.014 **	0.003 *	0.012 *	0.042 **	0.103 **	0.002 *
A_11_	−0.003	−0.006 **	−0.014 *	−0.027 **	−0.091 *	−0.0004
A_22_	−0.006	−0.003	−0.005	0.009	−0.071 *	−0.001
A_33_	−0.006	−0.002	0.002	0.001	0.003	−0.0003
A_12_	−0.012 *	−0.008 **	−0.015 *	−0.025 *	−0.107 *	−0.003 *
A_13_	−0.014 *	−0.0003	−0.015 *	−0.025 *	−0.114 *	−0.003 *
A_23_	−0.013 *	−0.001	−0.014 *	−0.033 *	−0.071 *	−0.003 *
Model	**	***	**	**	**	**
Lack of fit	ns	ns	ns	ns	ns	ns
R^2^	0.880	0.933	0.867	0.903	0.898	0.851
R^2^_adj_	0.772	0.873	0.748	0.816	0.806	0.716

ns, Not significant at *p* ≤ 0.05; * Significant at *p* ≤ 0.05; ** Significant at *p* ≤ 0.01; *** Significant at *p* ≤ 0.0001.

**Figure 8:**
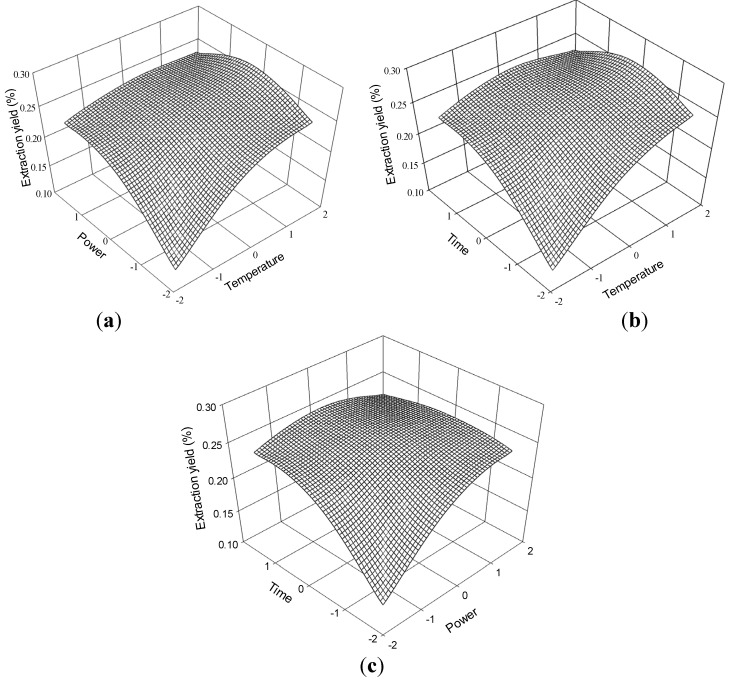
Response surface plots for the effects of (**a**) temperature/power (**b**) temperature/time (**c**) power/time on the extraction yield of ferulic acid.

The three-dimensional response surface plots in Figure 8 illustrate the relationship between the extraction yield of ferulic acid and the experimental variables. Figure 8a illustrates the interaction effect of temperature and power, when the time was set at its 0 level (70 min), on the extraction yield of ferulic acid. Ferulic acid yields gradually increased with temperature and ultrasonic power and peaked at approximately 70 °C to 80 °C and 175 W to 200 W. However, extraction yield of ferulic acid began to decrease after further increase in these parameters.

The interaction effects between temperature and time on the ferulic acid extraction yield when the power was set at its 0 level (175 W) are presented in Figure 8b. The yield of ferulic acid increased and peaked at 60 °C to 70 °C from 65 min to 75 min.

The interaction effects of power and time at 60 °C (0 levels) on the extraction yield of ferulic acid are presented in Figure 8c. Strong interaction was observed when the power was within 175 W to 200 W and the time ranged 65 min to 75 min, which contributed to the increased extraction yield.

The ferulic acid regression model for the statistical frequency method of analysis with 95% confidence interval was obtained (X_1_: 1.33 to 1.59, X_2_: -0.82 to 0.24 and X_3_: −0.65 to 0.38) when extraction yield >0.25% (*n* = 21). Therefore, the optimal conditions were 73.32 °C to 75.98 °C, 154.50 W to 181.03W, and 63.50 min to 73.77 min.

#### 2.2.2. Effect of process variables on the extraction yield of senkyunolide I

**Figure 9:**
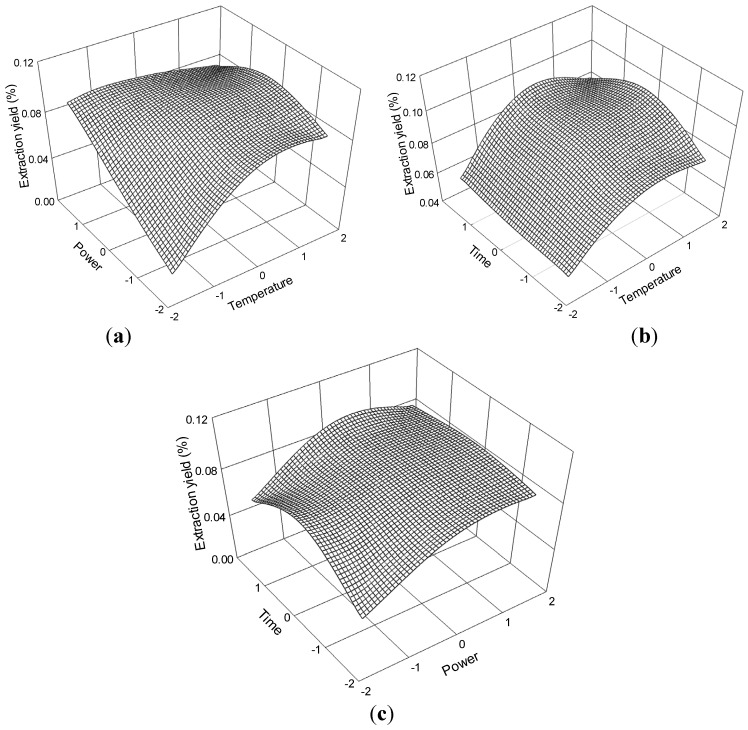
Response surface plots for the effects of (**a**) temperature/power (**b**) temperature/time (**c**) power/time on the extraction yield of senkyunolide I.

The senkyunolide I extraction yield results are presented in Table 2. The regression analysis results indicated that the main extraction parameters of senkyunolide I were temperature (X_1_), ultrasonic power (X_2_), and time (X_3_). The relationships between the extraction yield of senkyunolide I (Y_2_, Table 3) and the variables are shown in Figure 9. The contributions of extraction temperature, ultrasonic power, and extraction time were 2.40, 2.16 and 1.22, respectively. Extraction temperature had the greatest impact on the senkyunolide I extraction yield.

The effect of temperature and ultrasonic power on the extraction yield of senkyunolide I at constant time (0 levels) is shown in Figure 9a. The extraction yield of senkyunolide I gradually increased with temperature and power and peaked at 60 °C to 70 °C and 175 W to 200 W. Extraction yield of senkyunolide I began to decrease beyond 70 °C and 200 W. The appropriate extraction time (70 min) had positive effects on the extraction yield as shown in the response surface plots for the effect of time on extraction yield (Figure 9b,c) at constant ultrasonic power and constant temperature.

The senkyunolide I regression model for the statistical frequency method of analysis with 95% confidence interval was obtained (X_1_: 0.31 to 1.06, X_2_: −0.53 to 0.65 and X_3_: 0.60 to 1.30) when extraction yield > 0.09% (*n* = 23). Hence, the optimal conditions were 63.14 °C to 70.57 °C, 161.80 W to 191.18 W and 76.02 to 82.96 min.

#### 2.2.3. Effect of Process Variables on the Extraction Yield of Senkyunolide H

The extraction yield of senkyunolide H is presented in Table 2. Regression analysis showed that the extraction yield (Y_3_, Table 3) was significantly affected by the temperature (X_1_), ultrasonic power (X_2_), and time (X_3_), with corresponding contribution rates of 2.67, 1.75, and 1.67, respectively. Extraction temperature exhibited the greatest impact on the extraction yield of senkyunolide H.

**Figure 10:**
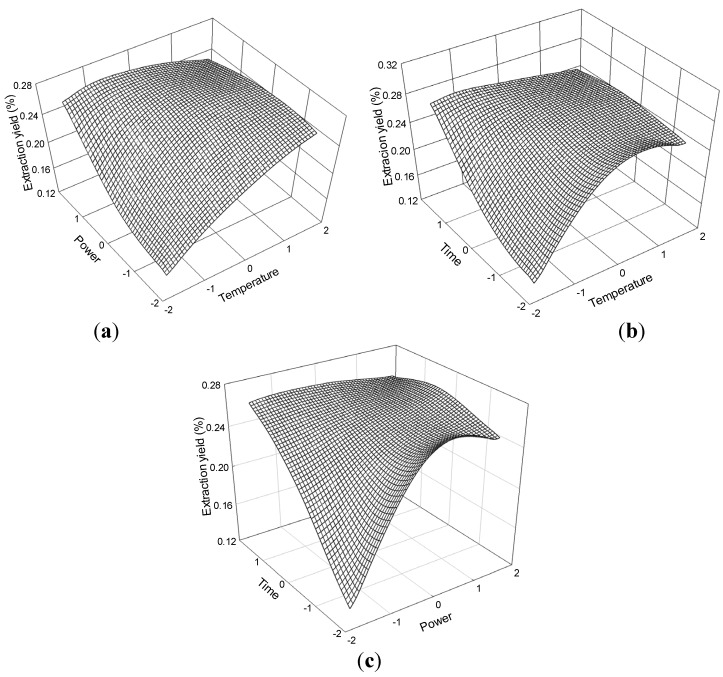
Response surface plots for the effects of (**a**) temperature/power (**b**) temperature/time (**c**) power/time on the extraction yield of senkyunolide H.

The relationship of the extraction yield of senkyunolide H and process variables are depicted in Figure 10. The effects of temperature and ultrasonic power on extraction yield at 0 level fixed time are shown in Figure 10a. The extraction yield of senkyunolide H gradually increased with temperature and power and peaked at approximately 70 °C to 75 °C and 175 W to 200 W. Further increases in these parameters resulted in decreased extraction yield of senkyunolide H. 

The response surface plots for the effect of time on extraction yield (Figure 10b,c) at constant ultrasonic power and constant temperature show that appropriate extraction time (60 min to 80 min) had positive effects on the extraction yield.

The senkyunolide H regression model for the statistical frequency method of analysis with 95% confidence interval was obtained (X_1_: 0.01 to 0.85, X_2_: −0.19 to 1.02, and X_3_: −0.30 to 0.94) when extraction yield > 0.26% (*n* = 23). Hence, the optimal conditions were 60.11 °C to 68.51 °C, 170.28 W to 200.50 W, and 77.05 min to 79.36 min.

#### 2.2.4. Effect of Process Variables on the Extraction Yield of Senkyunolide A

The extraction yield of senkyunolide A is presented in the Table 2. Regression analysis showed that the extraction yield (Y_4_, Table 3) was significantly affected by temperature (X_1_), ultrasonic power (X_2_), and time (X_3_), with corresponding contribution rates of 2.65, 2.05, and 1.81. Extraction temperature had the greatest impact on the extraction yield of senkyunolide A.

**Figure 11:**
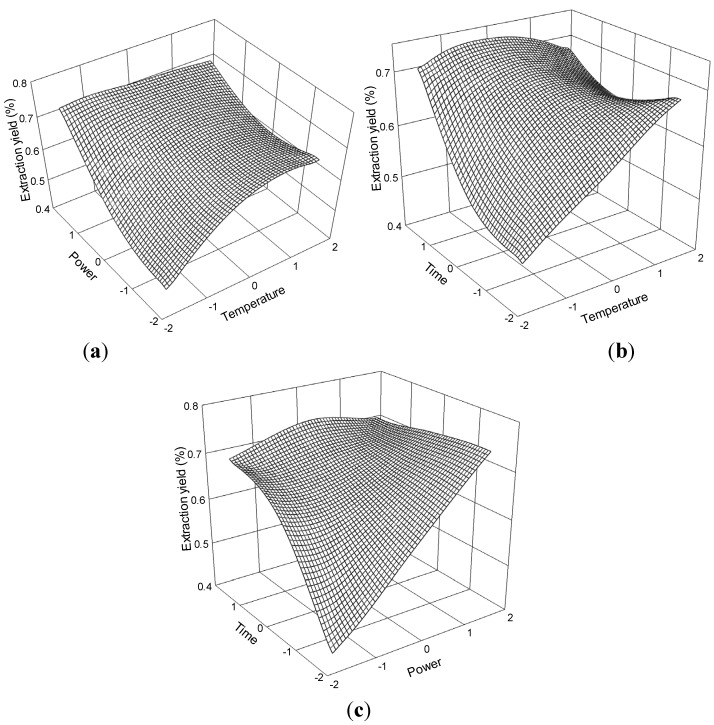
Response surface plots for the effects of (**a**) temperature/power (**b**) temperature/time (**c**) power/time on the extraction yield of senkyunolide A.

The relationship of the extraction yield of senkyunolide A and process variables are depicted in Figure 11. The effects of temperature and ultrasonic power on extraction yield at 0 level fixed time are shown in Figure 11a. 

Extraction yield gradually increased with temperature and power and peaked at approximately 70 °C to 75 °C and 175 W to 200 W. Further increases in these parameters resulted in decreased senkyunolide A extraction yields. The response surface plots for the effect of time on extraction yield (Figure 11b,c) at constant ultrasonic power and constant temperature showed that appropriate extraction time (60 min to 80 min) had positive effects on the extraction yield.

The senkyunolide A regression model for the statistical frequency method of analysis with 95% confidence interval was obtained (X_1_: −0.32 to 0.48, X_2_: −0.12 to 1.14, and X_3_: 0.14 to 1.24) when extraction yield >0.72% (*n* = 21). Hence, the optimal conditions were 56.80 °C to 64.80 °C, 172.08 W to 203.58 W, and 71.39 min to 82.38 min.

#### 2.2.5. Effect of Process Variables on the Extraction Yield of Ligustilide

Regression analysis was performed using the experimental data, and the model coefficients were evaluated for significance. Temperature (X_1_), ultrasonic power (X_2_), and time (X_3_) significantly affected the extraction yield of ligustilide (Y_5_, Table 3), with corresponding contribution rates of 2.72, 2.61, and 1.77. Extraction temperature showed the greatest impact on the extraction yield of ligustilide.

**Figure 12:**
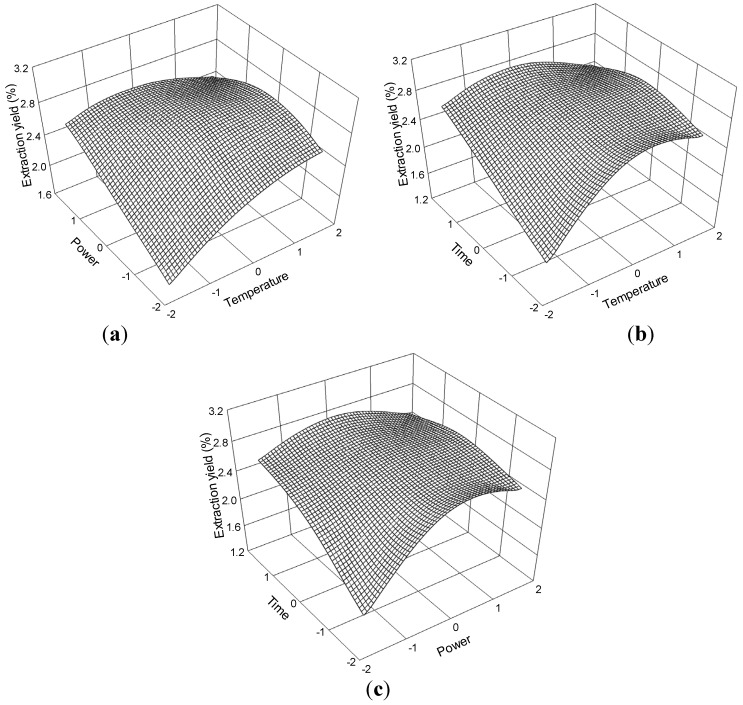
Response surface plots for the effects of (**a**) temperature/power (**b**) temperature/time (**c**) power/time on the extraction yield of ligustilide.

The three-dimensional response surface plots (Figure 12) illustrate the relationship between the extraction yield of ligustilide and experimental variables. These plots present the response as a function of two factors with another variable constant at its 0 level. Figure 12a shows the interaction effect between temperature and power, when the time was set at its 0 level (70 min) in the extraction of ligustilide. Extraction yield of ligustilide gradually increased with temperature and ultrasonic power and peaked at approximately 60 °C to 70 °C and 175 W.

The interaction effect between temperature and time at 0 level (175 W) power on the extraction of ligustilide is presented in Figure 12b. The response surface plot shows that the extraction yield of ligustilide increased and reached the maximum level at 60 °C to 70 °C for the time interval of 60 min to 80 min.

The interaction effect between power and time at 0 level (60 °C) temperature on the extraction yield of ligustilide is presented in Figure 12c. Strong interaction was observed when the power reached 175 W to 200 W and time reached 60 min to 80 min, which contributed to the increase in extraction yield.

The ligustilide regression model for the statistical frequency method of analysis with 95% confidence interval was obtained (X_1_: 0.41 to 0.48, X_2_: −0.12 to 0.71, and X_3_: −0.69 to 0.44) when extraction yield > 2.72% (*n* = 24). Hence, the optimal conditions were 64.14 °C to 70.37 °C, 171.98 W to 192.70 W, and 63.08 min to 74.42 min.

#### 2.2.6. Effect of Process Variables on the Extraction Yield of Levistolide A

The extraction yield of levistolide A is presented in the Table 2. Regression analysis showed that the extraction yield (Y_6_, Table 3) was significantly affected by temperature (X_1_), ultrasonic power (X_2_), and time (X_3_), with corresponding contribution rates of 1.78, 2.44, and 1.72. Ultrasonic power had the greatest impact on the extraction yield of levistolide A.

**Figure 13:**
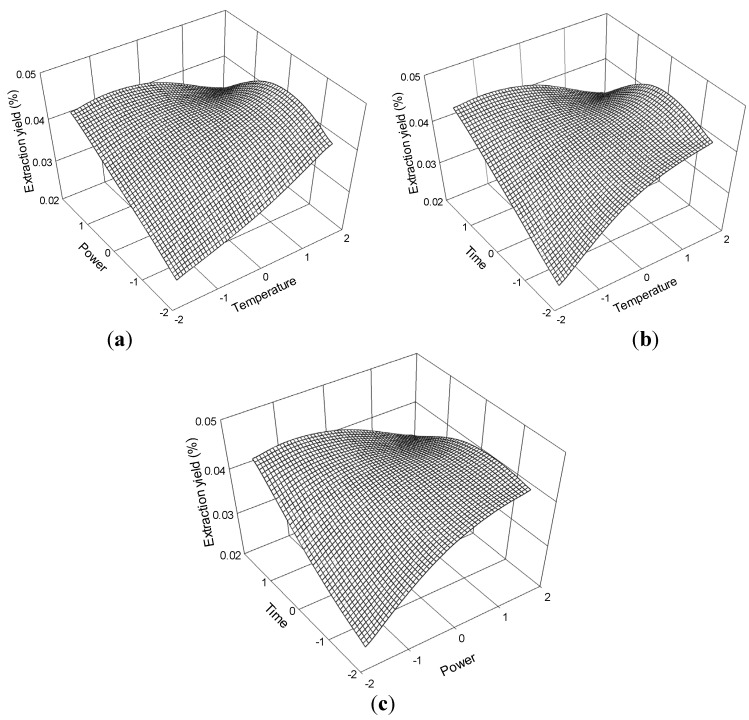
Response surface plots for the effects of (**a**) temperature/power (**b**) temperature/time (**c**) power/time on the extraction yield of levistolide A.

The relationship of the extraction yield of levistolide A and process variables are depicted in Figure 13. The effects of temperature and ultrasonic power on extraction yield at 0 level fixed time are shown in Figure 13a. Extraction yield of levistolide A gradually increased with temperature and power and peaked at about 70 °C to 80 °C and 175 W to 200 W. The response surface plots for the effect of time on extraction yield (Figure 13b,c) at constant ultrasonic power and constant temperature showed that, appropriate extraction time (60 min to 80 min) had positive effects on the extraction yield.

The levistolide A regression model for the statistical frequency method of analysis with 95% confidence interval was obtained (X_1_: 0.18 to 0.97, X_2_: 0.18 to 0.97, and X_3_: −0.99 to 0.21) when extraction yield > 0.04% (*n* = 22). Hence, the optimal conditions were 61.84 °C to 69.73 °C, 179.60 W to 199.33 W, and 60.01 min to 72.10 min.

### 2.3. Optimization of the Extraction Process

Table 4 indicates the optimum high-pressure ultrasound-assisted conditions for the extraction of the six major constituents from *L. chuanxiong* using RSM. Thirty six accurately weighed samples (0.5 g each filtered through 80 mesh sieve) were added to 50 mL 40% EtOH, pressurized 10 MPa, and divided into six groups. A set of samples was extracted under the optimum single ingredient conditions (obtained using statistical software), and the predicted results fitted well with the experimental results (Table 4).

**Table 4:** Estimated optimum conditions, predicted and experimental values of responses under these conditions.

Response variables	Optimum extraction conditions (obtained from equation)			Maximum extraction yields (%)		Extraction yields at optimal conditions from statistical frequency method (%)
	Temperature (°C)	Ultrasonic power (W)	Time (min)	Predicted	Actual	
Ferulic acid	77 (76.7)	195 (195.3)	64 (64.1)	0.27	0.27	0.27 ^ns^
Senkyunolide I	65 (65.3)	187 (186.8)	62 (61.5)	0.09	0.09	0.09 ^ns^
Senkyunolide H	60 (59.5)	205 (205.0)	75 (75.2)	0.26	0.27	0.27 ^ns^
Senkyunolide A	64 (63.6)	200 (200.4)	67 (66.6)	0.73	0.73	0.69 **
Ligustilide	66 (65.9)	184 (183.8)	71 (70.8)	2.74	2.76	2.75 ^ns^
Levistolide A	61 (61.4)	185 (185.0)	74 (74.2)	0.04	0.04	0.04 ^ns^

** Compared with the significant for actual yield of optimum conditions and statistical frequency conditions at *p* ≤ 0.01; ^ns^, Not significant at *p* ≤ 0.01 or *p* ≤ 0.05.

Based on the statistics frequency method, the following optimum extraction conditions were obtained: extraction temperature, 70 °C; ultrasonic power, 180 W; and extraction time, 74 min. Six accurately weighed samples (0.5 g each and filtered through 80 mesh sieve) were added to 50 mL 40% EtOH, pressurized 10 MPa, and extracted. The optimum extraction conditions were obtained through the statistics frequency method (Table 4). The calculated extraction yields of statistical frequency condition were compared with the actual extraction yields under optimum conditions. 

The results showed that the significant results were obtained in the extraction yields of senkyunolide A and ligustilide, contrary to the other four constituents, are not significant. Therefore, the optimum extraction conditions for simultaneous extraction of the six ingredients can be obtained through the statistical frequency method, and optimum extraction condition for one ingredient can be obtained through the single component method.

## 3. Experimental

### 3.1. General Information

Methanol (HPLC grade) and acetonitrile (HPLC grade) were purchased from Fisher Scientific, Inc. (Pittsburgh, PA, USA). Ethanol (HPLC grade) and acetic acid (AC grade) were purchased from Chengdu Kelong Chemical Factory (Chengdu, China). Water (HPLC grade) was purified through Milli-Q Plus system (Millipore, Bedford, MA, USA). Standard samples of ferulic acid, ligustilide and levistolide A were obtained from National Institutes for Food and Drug Control (Beijing, China). Senkyunolide I, senkyunolide H, and senkyunolide A were purchased from Beijing Beiyanxinglv Biotechnology Co., Ltd (Beijing, China). *L. chuanxiong* Hort. rhizomes were collected from the experimental field in Dujiangyan County, Chengdu City (Sichuan Province, China) during harvest time. The materials were confirmed based on morphologic, microscopic, and physiochemical analyses according to the Chinese Pharmacopoeia [1]. Voucher specimens were deposited in the College of Agronomy, Sichuan Agricultural University. Samples were sun-dried and ground into powder.

### 3.2. HPLC Quantitative Analysis of the Six Major Constituents

HPLC analysis was performed on Agilent 1200 series HPLC-DAD system comprising a vacuum degasser, quaternary pump, autosampler, thermostated column compartment and diode array detector (Agilent, Palo Alto, CA, USA) to analyze the samples extracts. The extracts (10 μL) were injected and separated on a Symmetry C_18_ column (250 mm × 4.6 mm, 5 μm, Waters, Milford, MA, USA). The mobile phase consisted of methanol (A), acetonitrile (B), and 1% aqueous acetic acid (C) using a gradient program of 40: 20: 40 (A: B: C, v/v/v) at 0 min to 5 min, 60% to 100% (B), and 40% to 0% (C) from 5 min to 30 min. The flow rate was 1 mL/min, and the column temperature was 35 °C. The detection wavelength was set to 0 min to 4.3 min 321 nm and to 275 nm from 4.31 min to 30 min.

### 3.3. Single Factor Tests

To investigate the extraction of the six major constituents under different conditions, single factor tests were employed first to determine the optimal extraction solvent, pressure, particle size, liquid-to-solid ratio, extraction temperature, ultrasonic power and extraction time. The six major ingredients of *L. chuanxiong* rhizome were extracted using a SB-5200 DTD ultrasonic extractor (Ningbo Science Biotechnology Co., Ltd, Zhejiang, China). The author-customized high-pressure extraction tubes containing the sample extract were then partially immersed in the ultrasonic bath, and the liquid in the tubes was kept at the same level as that of the water in the bath. Finally, each extracted solution was filtered through a 0.45 μm syringe filter and collected into a 1.5 mL volume vial.

#### 3.3.1. Solvent Selection

Each extract (0.5 g of dried powder filtered through 60 mesh) was mixed with extraction solvent (25 mL of 100% MeOH, 80% MeOH, 60% MeOH, 40% MeOH, 20% MeOH, 100% EtOH, 80% EtOH, 60% EtOH, 40% EtOH or 20% EtOH) in a 50 mL-volume tube and pressurized to 10 MPa. The working frequency was 40 kHz, ultrasonic power rating was 200 W, temperature was 40 °C, and the extraction time was 60 min. The experiments were conducted in triplicate (30 experimental treatments) and extraction yield was expressed as a percentage using following equation: extraction yield (%) = ingredient weight/sample weight × 100. 

#### 3.3.2. Pressure Selection

Each extract (0.5 g of dried powder was filtered through 60 mesh) was dissolved in 40% EtOH (25 mL, based on the results from Section 3.3.1.) and prepared for seven levels of pressure treatment (−0.05, 0.1, 2, 4, 6, 8, and 10 MPa). The experiments were performed in triplicate (21 experimental treatments). The extraction conditions were as follows: working frequency, 40 kHz; ultrasonic power, 200 W; temperature, 40 °C; and extraction time, 60 min.

#### 3.3.3. Particle Size Selection

Each extract [0.5 g of dried powder filtered through different meshes (20, 40, 60, 80, and 100 mesh)] was mixed with 40% EtOH (25 mL) and pressurized to 10 MPa (based on the results from Section 3.3.2.). The experiments were conducted in triplicate (15 experimental treatments). The extraction conditions were as follows: working frequency, 40 kHz; ultrasonic power, 200 W; temperature, 40 °C, and extraction time, 60 min.

#### 3.3.4. Liquid-to-Solid Ratio Selection

Each extract [0.5 g of dried powder (filtered through 80 mesh based on the results of Section 3.3.3.)] was dissolved in different volumes of 40% EtOH (100, 50, 40, 30, 20 and 10 mL) and pressurized to 10 MPa. The experiments were conducted in triplicate (18 experimental treatments). The working frequency was 40 kHz, ultrasonic power was 200 W, temperature was 40 °C, and extraction time was 60 min.

#### 3.3.5. Extraction Temperature Selection

Each extract (0.5 g of dried powder) was mixed 40% EtOH (50 mL, based on the results from Section 3.3.4.) and pressurized to 10 MPa. The experiments were conducted at different extraction temperatures of 20, 30, 40, 50, 60, and 70 °C. The working ultrasonic power and time were 200 W and 60 min, respectively. The experiments were also performed in triplicate (18 experimental treatments).

#### 3.3.6. Ultrasonic Power Selection

Each extract (0.5 g of dried powder) was mixed 40% EtOH (50 mL) and pressurized to 10 MPa. Different ultrasonic power levels of 75, 100, 125, 150, 175, 200, 225, and 250 W were applied. The working temperature and time were 40 °C and 60 min, respectively. The experiments were performed in triplicate (24 experimental treatments).

#### 3.3.7. Extraction Time Selection

Each extract (0.5 g of dried powder) was mixed 40% EtOH (50 mL) and pressurized to 10 MPa. The working ultrasonic power and temperature were 200 W and 40 °C, respectively. The extractions were performed for 30, 40, 50, 60, 70, 80, and 90 min. The experiments were conducted in triplicate (21 experimental treatments).

### 3.4. Central Composite Design

The single factor tests demonstrated the marked interaction among extraction temperature, ultrasonic power, and extraction time. Hence, RSM was used to investigate the influence of these three independent variables on the six extracts. The experiments were performed according to a rotatable central composite design (CCD) [46]. The coded values of the experimental factors and factor levels (Table 5) were used in the response surface analysis that was run 20× (Table 2) and performed in triplicate. The results of the response surface design were analyzed using Design Expert Software, version 8.0 (Stat-Ease, Inc., Minneapolis, MN, USA), and the contribution rate was calculated using the following equation:

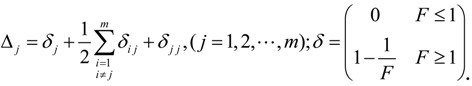

where, Δ*_j_* was the contribution rate; *F* were the *F* value for the linear effect terms, the interaction effect terms, and the quadratic effect terms (regression coefficients significance test; if using *F* test, direct calculation; if using *t*-test, *F_j_* = *t_j_^2^*, *F_ij_* = *t_ij_^2^*, and *F_jj_* = *t_jj_^2^*), respectively; *δ_j_*, *δ_ij_*, and *δ_jj_* were calculated *F* value of the independent variables, the interaction effect terms, and the quadratic effect terms, respectively. 

**Table 5:** Levels of variables for the experimental design.

Independent variables	Units	Range and Level				
		1.682(α)	1	0	−1	1.682(−α)
Extraction Temperature (X_1_)	°C	77	70	60	50	43
Ultrasonic Power (X_2_)	W	217.5	200	175	150	132.5
Extraction Time (X_3_)	min	87	80	70	60	53

## 4. Conclusions

In this study, RSM was successfully applied to optimize the high-pressure ultrasonic-assisted extraction of the six major constituents from *L. chuanxiong* rhizome. Extraction solvent type, pressure, sample particle size, liquid-to-solid ratio, extraction temperature, ultrasonic power, and extraction time played significant roles in the extraction of constituents. We were able to extract higher yields of the constituents from *L. chuanxiong*, while using a green extraction solvent, less solvent, lower extraction temperature, while simultaneously reducing considerably the extraction time. According to the statistical frequency method the optimum extraction conditions for the simultaneous extraction of the six components were 40% EtOH, 10 MPa, 80 mesh, 100: 1, 70 °C, 180 W, and 74 min. 
